# Advances in Pediatric Critical Care Research in India

**DOI:** 10.3389/fped.2018.00150

**Published:** 2018-05-28

**Authors:** Utpal Bhalala, Arun Bansal, Krishan Chugh

**Affiliations:** ^1^Baylor College of Medicine at The Children's Hospital of San Antonio, San Antonio, TX, United States; ^2^Postgraduate Institute of Medical Education and Research, Chandigarh, India; ^3^Fortis Hospital, New Delhi, India

**Keywords:** research, pediatric critical care, India, scientific evidence, advances

## Abstract

Over last 2 decades, there has been a significant progress made in the field of pediatric critical care in India. There has been complementary and parallel growth in the pediatric critical care services in India and the number of pediatric critical care providers who are either formally trained in India or who have returned to India after their formal training abroad. The pediatric critical care community in India has recognized obvious differences in profiles of critical illnesses and patients between Indian subcontinent and the West. Therefore there is a growing interest in generating scientific evidence through local research which would be applicable to critically ill children in Indian subcontinent. This article focuses on advances in pediatric critical care research in India and its future directions.

## Introduction

Pediatric Critical Care Medicine (PCCM) is a relatively new but a rapidly growing pediatric specialty in resource-limited countries. Pediatric Intensive Care Units (PICUs) were introduced into the lower- and middle-income countries (LMICs) somewhere in late 1980's or early 1990's. Few countries in Africa and south-east Asia still do not have a dedicated PICU. Though pediatric intensive care practices were introduced into India in the 1980's, early units were simply located in a special “treatment room” and lacked the constant monitoring of vital sign, respiratory or hemodynamic support characteristic of a PICU. One author (KC) recalls his first exposure to a PICU at Kalawati Saran Children's Hospital, Lady Hardinge Medical College in 1983 in New Delhi, although the first organized PICU was reportedly established in 1991 at Kanchi Kamakoti Childs Trust Hospital in Chennai, India with seven beds, a separate team of doctors and nurses, and with a pediatric anaesthesiologist as the director of the unit (1). Currently, there are more than 100 dedicated PICUs in the private and public sectors in India. With growth in pediatric critical care services in India, there has been a parallel growth in academic medicine in pediatric criticalc care in India. This article describes advances in critical care research in the west and in India, particularly in PCCM and future of pediatric critical care research in India.

### Advances in pediatric critical care research in the west

For any specialty in medicine, research is key to understanding the epidemiology and pathophysiology of disease processes, exploring potential new therapies and measuring response to therapies. With rapidly growing specialty of pediatric critical care, there have been parallel advances in research in pediatric critical care in the West. The first collaborative pediatric critical care study group (PCCSG) was founded by Gregory Stidham and associates in the early 1990s. Approximately 60 pediatric ICUs, mostly from the United States worked together to generate a number of studies related to PICU outcomes (2–7). The Pediatric Acute Lung Injury and Sepsis Investigators (PALISI) Network, was founded by Adrienne Randolph at Children's Hospital of Boston in the late 1990s. Around 48 pediatric ICUs throughout North America participated to study therapies for acute lung injury, sepsis and multi-organ failure (8–11). Randall Wetzel at Children's Hospital Los Angeles founded the first electronic database, the Virtual PICU (vPICU) in 2000, to create a shared patient database for outcomes analysis and improve critical care practices (12). In 2004, National Institutes of Health, founded the Collaborative Pediatric Critical Care Research Network (CPCCRN) to study the pathophysiological bases of critical illness and safety and efficacy of treatment of critically ill children (12). The pediatric interest group of Canadian Critical Care Trial Group (CCCTG) has conducted large, multicenter trials such as TRIPICU and HypHIT (13). Similarly, European Society of Pediatric and Neonatal Intensive Care (ESPNIC) has been active in multi-center evaluation of respiratory distress syndrome (14) and in collaboration with European Extracorporeal Life Support (EuroELSO), it has created a working group to evaluate actual and future trends about neonatal and pediatric ECMO in Europe (15). The ANZICS (Australian and New Zealand Intensive Care Society) Pediatric Group has fostered and promoted meaningful research within the Australian and New Zealand pediatric intensive care communities (16).

There are now innumerable, ongoing single-site and multi-site clinical trials and large database studies in America and Europe which are substantially contributing to our understanding of science of PCCM. Some examples of recent key studies are—therapeutic hypothermia after pediatric cardiac arrest (THAPCA), heart and lung failure—pediatric insulin titration (HALF-PINT) and randomized evaluation of sedation titration for respiratory failure (RESTORE) trials (17–19).

### Advances in adult critical care medicine research in India

Before the turn of the century, critical care medicine in India was practiced in a few hospitals, either by those interested in the field and learning on the job, or by physicians returning to India after training overseas. Establishment of the Indian Society of Critical Care Medicine (ISCCM) in 1993 was a turning point in the history of critical care medicine in India. ISCCM has grown to become one of the largest professionl organizations and serves as the premier organization supporting the training and research in critical care medicine in India (20). In 2012, the Medical Council of India (MCI) recognized critical care medicine as an independent speciality, enabling 3-year training programmes, which have facilitated the development of academic departments of intensive care medicine with greater emphasis on research (21). Adult critical care community in India has made significant contributions to large multicenter randomized controlled trials such as the PROWESS-SHOCK (22) and OSCILLATE studies (23) and to a number of international, multicenter observational studies (24–28). Over last decade, several multicenter, adult critical care studies conducted in ICUs in India have contributed to a body of literature relevant to the Indian population (21). Though, Divatia and co-authors reported a 35% increase in the number of abstracts submitted to the annual conference of the ISCCM in 2013 as compared to the 2012 conference, their publication rate in international journals has been low (21, 29). Time constraints and lack of motivation to publish could be important contributing factors to the low publication rate. Overall, the advances in adult critical care medicine research in India have encouraged the pediatric critical care community to make forward steps toward pediatric critical research relevant to the unique diseases and child populations in India.

### Advances in pediatric critical care research in India

The initiative to upgrade neonatal and pediatric critical care facilities has come primarily from major teaching institutions (30). India has shown tremendous growth in the practice of pediatric critical care. From a single PICU in 1991 to a young dynamic speciality in 2017, it has come a long way. India, foremost amongst the resource-limited countries has been a leader in that. The intensive care chapter of the Indian Academy of Pediatrics (IAP) started a formal fellowship training program in 2002 and they now have 22 accredited centers that are running this program successfully. More than 250 students have been trained through this program (31).

Similarly, research in neonatal and pediatric critical care has also picked up in India. The early published reports on outcomes of intensive care units in India were related to neonatal intensive care unit (NICU) graduates (32). Subsequently, Singhi and co-authors published the first report on electrolyte abnormalities in pediatric pneumonia in 1992 (33). As listed in PubMed, pediatric critical care research has grown from only 31 published clinical trials, 19 randomized controlled trials (RCTs) and 2 multicenter studies in 1990 to an impressive 198 RCTs and 92 multicenter studies in 2016. This progress shows that the growth of research in Pediatric Critical Care in India has come a long way. As the interest in research grew, the Government of India also tried to streamline it by setting up the Clinical Trial Registry of India (CTRI) in 2007. Registration of trial in CTRI is now mandatory and is also an important factor for publication in various journals. As on 30th June 2017, 8950 trials are registered with the CTRI (34).

Retrospective and prospective pediatric critical care studies conducted in India and their subsequent publication in regional and international journals have grown steadily in the past two decades (35–41). A handful of studies conducted by pediatric critical care researchers in India have impacted PICU care not only in the region but also outside the region—for example, original studies published from India on monitoring of intracranial and cerebral perfusion pressures in patients with acute meningoencephalitis have reinvigorated the field of neurocritical care (42–44). Recently, newer developments in collaborative, multicenter research initiatives in pediatric critical care have occurred in India. The Postgraduate Institute of Medical Education and Research (PGIMER) in Chandigarh, India has initiated the first, national, multi-institutional database for pediatric cardiac arrest and cardiopulmonary resuscitation. Currently, large, government-run, teaching institutions are putting collaborative efforts to launch similar multicenter collaborative studies.

**Figure 1:**
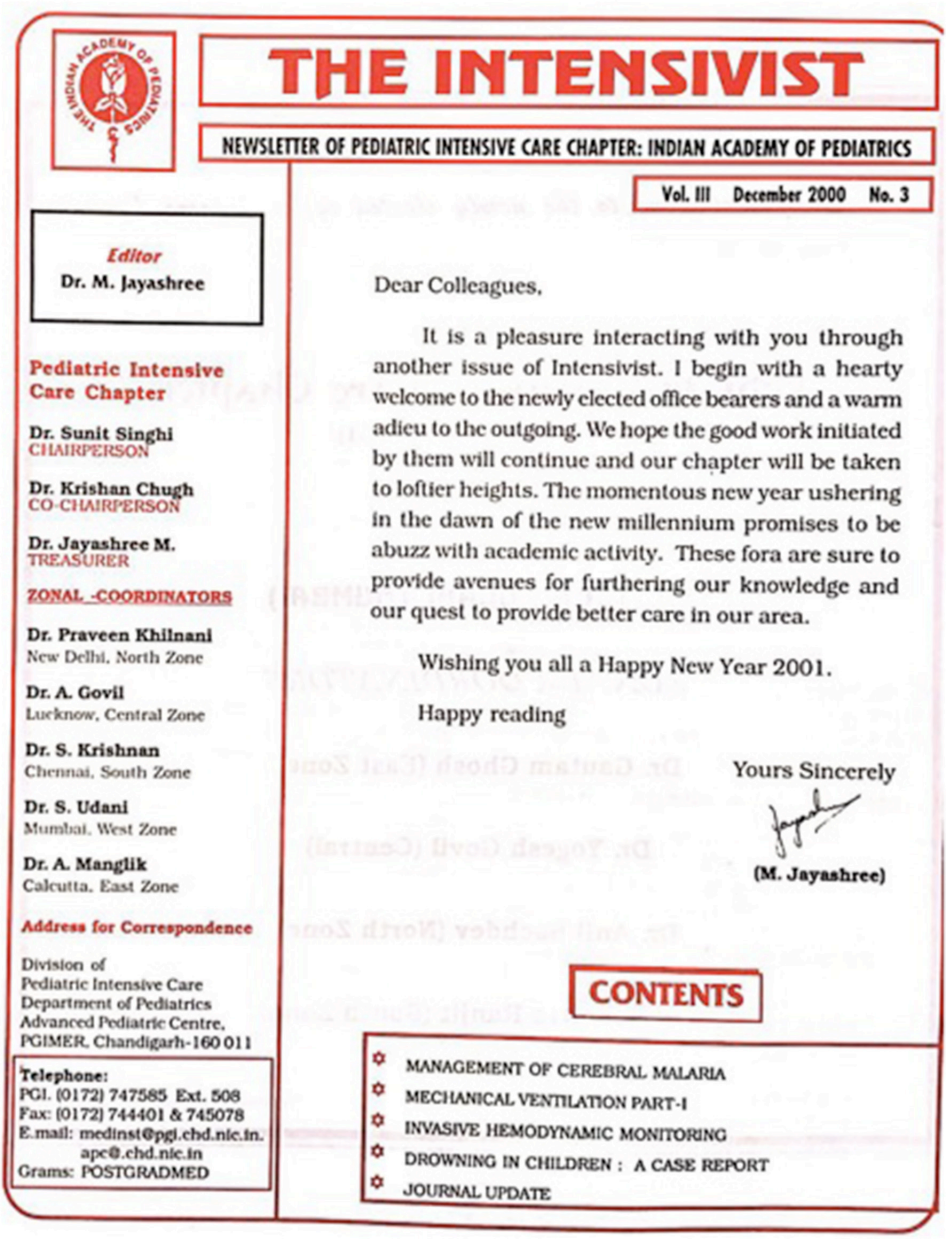
The picture of the periodical “The Intensivist” which was started by Indian Academy of Pediatrics Intensive Care Chapter, which ultimately culminated into launching of the official journal of pediatric critical care of the intensive care chapter of the Indian Academy of Pediatrics.

**Figure 2:**
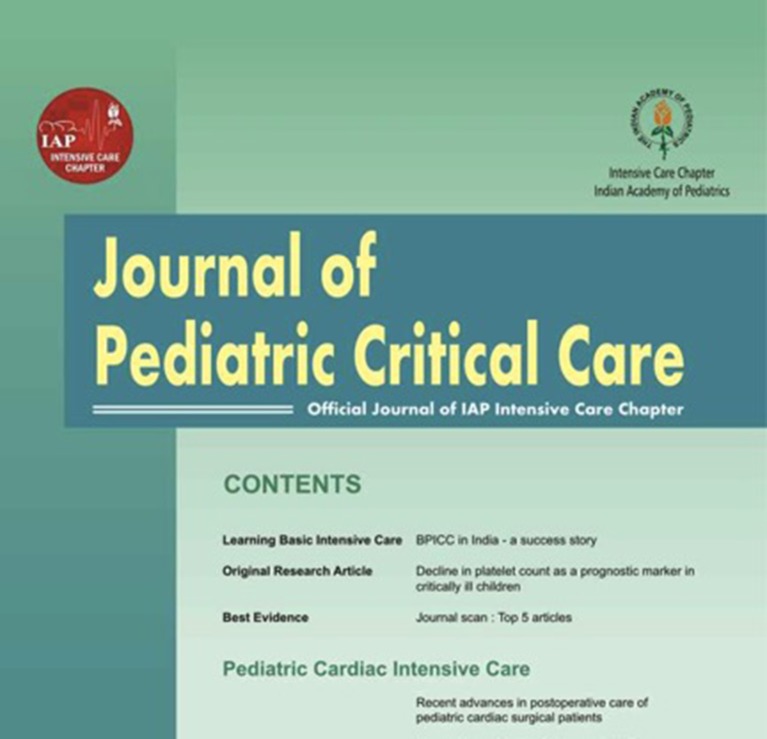
The picture of journal of pediatric critical care—the official journal of the intensive care chapter of the Indian Academy of Pediatrics.

Under the auspices of ISCCM, several pediatric critical care leaders in India have started a multicenter PICU data registry called INSPIRED (personal communication). Also, several institutions in India are now contributing to multicenter, international, pediatric critical care studies such as point prevalence studies, or quality improvement studies led by western institutions (45, 46). The Intensive Care Chapter of IAP started the quarterly periodical called “The Intensivist” (Figure 1), which grew in popularity over time and culminated into launching the Journal of Pediatric Critical Care in 2014, which is the official journal of the chapter (Figure 2) (47).

### Factors influencing advancement of pediatric critical care research in India

India is a large country and continues to be second most populated country in the world. The healthcare in India has been constantly challenged with issues of manpower, resources, infrastructure and financial support. Though the number of pediatric intensivists trained either within or outside India has grown significantly in India over last 15 years, current manpower is not sufficient to cope with the number of critically ill children in India. Due to heavy clinical commitments and burn-out issues, pediatric critical care professionals in India lack sufficient protected time for research. Also, unlike western countries, India has very limited government funds allocated to research. This further restricts the support needed for research data gathering, statistical analyses, research coordination and dissemination of research findings. Several research institutes, research task forces, and grants-in-aid developed by Indian Council of Medical Research (ICMR) have led to improved research support in India (48). The pediatric critical care faculty at large, government-run, teaching institutes have tapped into these resources for investigator-initiated clinical trials in PCCM. Also, some of the industry funds have also become available for PCCM research in India (49).

The initiation of PCCM fellowship training programs by the Intensive Care Chapter of IAP have not only promoted clinical training in pediatric critical care but also fueled research enthusiasm among young and budding pediatric intensivists in India (31). A formal 3-year post-doctoral training program in PCCM, started by the PGIMER in Chandigarh, India and subsequently adopted by two other premier institutes in India has paved the pathway for protected research time and formal research training of PICU fellows.

The national conference of pediatric critical care (NCPCC) organized annually by IAP intensive care chapter has provided tremendous opportunities to PCCM community in India to showcase and discuss their research findings and collaborate on research (50). Similarly, the annual conferences organized by World Federation of Pediatric Intensive and Critical Care Societies and Society of Critical Care Medicine have allowed networking of pediatric intensivists from India with those from other countries and improved research collaborations (51). Several global health institutions of leading universities and hospitals in the West have collaborated with institutes in India and conducted research related to neonatal and pediatric critical care (52).

### Future of pediatric critical care medicine research in India

India is a country with the world's second largest population, a relatively low median age, and a demographic trend that is very different from developed countries. Also India is a fast growing emerging market, especially in the field of information technology. This signifies a great potential for research especially in pediatrics given the young population and disease burden. It is likely that with a transition from a developing nation to a developed nation, India will witness a wide spectrum of healthcare issues of children common to both developing and developed nations. It is likely that there will be a growing population of longer-term survivors of prematurity, congenital heart disease and genetic syndromes, which used to be otherwise deemed fatal in the past. These future trends will affect PICU research in India.

As the PCCM specialty continues to grow, more research is likely to occur at both government-run, teaching institutions as well as corporate hospitals. Due to several obvious differences in disease profiles, host characteristics (e.g., malnutrition), access to medical care, or resource availability and allocation, the pediatric critical care community understands that scientific evidence generated through studies done in the West cannot always be extrapolated to patients in the Indian context. Among the pediatric intensivists, there is a growing awareness of the need of generating scientific evidence locally through studies within India. This is likely to culminate into an increase in investigator-initiated, single-center studies and also improved collaboration among a larger number of PCCM programs to create data registries and multicenter clinical trials in India.

The strengths of critical care medicine community in India include a huge knowledge-base for tropical and infectious diseases (53, 54), large patient burden, cost-effective strategies and the development of low-cost technology solutions through frugal innovation (55). As the investigator-initiated single-center and multicenters studies in PCCM grow in India, parallel efforts need to be made by government and corporate healthcare systems to improve the research infrastructure. Also, It will be equally important for institutions and professional organizations such as the ISCCM and IAP to establish structured research training for PCCM in India. A quality improvement research project and/or research thesis done during fellowship and an opportunity to present research work at a national and international conferences might encourage the pediatric critical care fellows to pursue academic careers. Finally, the contributions of national organizations such as ISCCM in supporting research through research reources, funding and research collaborations will define the future of the pediatric critical care research in India.

## Author contributions

All authors listed have made a substantial, direct and intellectual contribution to the work, and approved it for publication.

## Conflict of interest statement

The authors declare that the research was conducted in the absence of any commercial or financial relationships that could be construed as a potential conflict of interest.
